# Obstetric Aphorisms for the Use of Students Commencing Midwifery Practice

**Published:** 1914-03

**Authors:** 


**Obstetric Aphorisms for the use of Students commencing Midwifery Practice.** By JOSEPH GRIFFITHS SWAYNE, M.D. Eleventh Edition, revised and edited by WALTER CARLESS SWAYNE, M.D. Pp. xvi, 216. London: J. & A. Churchill. 1913. Price 3s. 6d. net.

Swayne's *Obstetric Aphorisms* was well known as an almost indispensable companion to students taking out practical midwifery long before 1893, the date affixed to what is in this edition stated to be the preface to the first. In that preface Professor Joseph Swayne said that "the object of this work is to give the student a few brief and practical directions respecting the management of ordinary cases of labour, and also to point out to him, in extraordinary cases, when and how he may act upon his own responsibility, and when he ought to send for assistance. The student is never placed in a more trying situation, nor has to incur a greater amount of responsibility, than when he is attending a difficult case of labour in a place remote from medical aid; and the end of this work will be fully answered if it serve to keep any, who may be so situated, from the opposite extremes of temerity or timidity. It is not intended to be used in any way as a substitute for a systematic treatise on midwifery."

The work has for some years been out of print, and we know that students have felt the want of such a guide as this, and they will no doubt feel grateful to Professor Walter Swayne for having supplied that want. In revising and bringing this new edition up to date, the editor tells us that he "found it difficult to do justice to such a work and yet retain its original character," and that he has endeavoured "to retain the experience and sound judgment of the author even at the expense of modernity." In this he has been successful even to a fault; for we think that he might have employed the pruning-knife more freely: such terms, for example, as milk, ephemeral, and miliary, applied to fevers, might have been deleted. Upon the whole, however, the work has been thoroughly revised and brought up to date, and is admirably suited to the purpose for which it is intended. Every student taking out practical midwifery should carry a copy in his pocket, and refer to it in all cases of doubt or difficulty. He will then understand how to manage ordinary cases, what deviations from the normal he may undertake by himself, and when he should send for assistance, in fact all that he needs to know.

